# Supplementary material for: Cloud BioLinux: pre-configured and on-demand bioinformatics computing for the genomics community
Source: BMC Bioinformatics. 2012 Mar 19;13:42. doi: 10.1186/1471-2105-13-42 (PMC3372431; doi:10.1186/1471-2105-13-42)
Supplement: Additional file 1 — Supplementary 1 Cloud BioLinux software documentation in the form of a mini, self-contained website. Users need to download and uncompress the .zip file, and open through a web browser the "index.html" file available on the main directory. (ZIP 1823 kb). [file 1471-2105-13-42-S1.ZIP › Cloud-BioLinux-Package-Documentation/docs/protdist.html]

Bio-Linux Software Documentation Pages

Back to search form

## protdist

|  |  |
| --- | --- |
| Name | protdist |
| Description | **protdist** is part of the PHYLIP package.  Copyright 1993, 2000-2004 by the University of Washington. Permission is granted to copy this document provided that no fee is charged for it and that this copyright notice is not removed. This program uses protein sequences to compute a distance matrix, under four different models of amino acid replacement. It can also compute a table of similarity between the amino acid sequences. The distance for each pair of species estimates the total branch length between the two species, and can be used in the distance matrix programs **fitch**, **kitsch** or **neighbor**.  This is an alternative to use of the sequence data itself in the parsimony program PROTPARS.  The program reads in protein sequences and writes an output file containing the distance matrix or similarity table. The five models of amino acid substitution are one which is based on the Jones, Taylor and Thornton (1992) model of amino acid change, the PMB model (Veerassamy, Smith and Tillier, 2004) which is derived from the Blocks database of conserved protein motifs, one based on the PAM matrixes of Margaret Dayhoff, one due to Kimura (1983) which approximates it based simply on the fraction of similar amino acids, and one based on a model in which the amino acids are divided up into groups, with change occurring based on the genetic code but with greater difficulty of changing between groups. The program correctly takes into account a variety of sequence ambiguities.  **References:**  Felsenstein, J. 1993. PHYLIP (Phylogeny Inference Package) version 3.5c. Distributed by the author. Department of Genetics, University of Washington, Seattle.    Felsenstein, J. 1989. PHYLIP -- Phylogeny Inference Package (Version 3.2). Cladistics 5: 164-166. |
| Homepage | http://evolution.genetics.washington.edu/phylip.html |
| Remote Documentation | http://evolution.genetics.washington.edu/phylip/doc/protdist.html |
